# Origin of the insulating phase and metal-insulator transition in the organic molecular solid *κ*-(BEDT-TTF)_2_Cu_2_(CN)_3_

**DOI:** 10.1038/s41524-026-01960-y

**Published:** 2026-01-17

**Authors:** Dongbin Shin, Fabijan Pavošević, Nicolas Tancogne-Dejean, Michele Buzzi, Emil Viñas Boström, Angel Rubio

**Affiliations:** 1 Department of Physics and Photon Science, Gwangju Institute of Science and Technology (GIST), Gwangju, Republic of Korea; 2 Max Planck Institute for the Structure and Dynamics of Matter, Hamburg, Germany; 3 Algorithmiq Ltd., Helsinki, Finland; 4 Nano-Bio Spectroscopy Group, Departamento de Física de Materiales, Universidad del País Vasco, San Sebastian, Spain; 5 Initiative for Computational Catalysis (ICC), The Flatiron Institute, New York, NY USA

**Keywords:** Materials science, Physics

## Abstract

Recent studies of organic molecular solids have focused on their complex phase diagram and on light-induced phenomena, including a Mott insulating state, a spin liquid phase, and light-enhanced superconductivity. However, discrepancies between experiments and first-principles calculations for the *κ*-(BEDT-TTF)_2_X family hinder a comprehensive understanding of their properties. Here, we revisit the electronic structure of *κ*-(BEDT-TTF)_2_Cu_2_(CN)_3_ with a recently developed method for applying the Hubbard *U* potential on generalized orbital states, within the framework of density functional theory, to correct the orbital energy levels of the molecular solid. Our work focuses on the electronic structure of *κ*-(BEDT-TTF)_2_Cu_2_(CN)_3_, whose insulating state originates from an energy gap between the highest occupied and the lowest unoccupied molecular orbital states of the BEDT-TTF dimers, which constitute the periodic unit of the molecular solid. Our calculations provide results in alignment with experiments for band gaps, optical conductivities, and evolution of the metal-insulator transition as a function of pressure. Especially, the observed superconducting dome of *κ*-(BEDT-TTF)_2_Cu_2_(CN)_3_, which derives from the flat band state at the Fermi level, is qualitatively reproduced. Additionally, we construct a new low-energy lattice model based on our first-principles computed band structure that can be exploited to address many-body physics, such as quantum spin liquid states and double-holon dynamics. Our work can be extended to achieve deeper insight into the complex phase diagram and light-induced phenomena in the *κ*-(BEDT-TTF)_2_X family and other complex organic molecular solids.

## Introduction

Molecular solids have attracted significant attention in recent years due to their complex phase diagrams and exceptional light-induced phenomena^1–8^. For instance, *κ*-(BEDT-TTF)_2_Cu_2_(CN)_3_ (*κ*-ET-CN) exhibits an antiferromagnetic Mott insulating electronic structure under ambient conditions but shows a metal-insulator transition under moderate pressure, with critical pressure of 0.37 GPa, ultimately leading to a superconducting phase^1,8–10^. It is a potential candidate to exhibit a quantum spin liquid phase, characterized by long-range spin entanglement and magnetic frustration due to quantum fluctuations, while also exhibiting light-enhanced superconductivity and ultrafast gap dynamics induced by light^4–7,11–15^. A simple two-band lattice mode (tight-binding (TB) Hamiltonian) is usually employed to investigate the many-body correlations in the *κ*-(BEDT-TTF)_2_X (*κ*-ET) family, but the metal-insulator transition induced by external pressure remains poorly understood from first-principle calculations, which usually lack a detailed description of electron-electron correlation effects^16,17^. On the other hand, a similar phase diagram is observed in other molecular solids, like alkali-doped C_60_, whose antiferromagnetic insulating and superconducting phases under pressure are described using advanced methods such as dynamical mean-field theory (DMFT) combined with density functional theory (DFT)^3,18,19^. These reports suggest that an advanced theoretical approach is necessary to elucidate the microscopic mechanism behind the intriguing phenomena in these molecular solids.

Recent developments in the DFT’s description of exchange and correlation effects have accelerated the ability to evaluate accurate electronic structures of complex correlated condensed matter systems^20–23^. Conventional DFT exchange-correlation approximations effectively allow the study of materials in condensed matter physics from a first-principles approach^24^, but often fail to accurately capture the electronic structure of correlated systems like metal-oxides and organic molecular solids^18,20,25,26^. This failure usually originates from the delocalized nature of the adiabatic density functional approximation, where the electron-electron exchange potential is represented in terms of charge density rather than state interactions^27–29^. Extended approaches to DFT have been developed to overcome this limitation, such as hybrid functionals, DFT+DMFT, and auxiliary field quantum Monte Carlo^21,22,30,31^. While these approaches provide accurate values of band gaps in solids and energy levels in molecular systems, consistent with experimental observations^21,32^, their high computational cost limits their application to condensed matter systems. In contrast to the above approaches, the DFT plus Hubbard U (DFT+U) method makes it possible to correct the on-site Coulomb interaction of atomic orbitals at a smaller computational cost^20,33,34^, and the DFT+U+V method adds inter-atomic corrections (V) to further improve optical response accuracy in metal oxides^35–38^. In addition, the recently introduced method combining DFT with a Hubbard U potential on generalized orbital states (DFT+GOU), an extension of DFT+U, improves the description of correlated systems by correcting the on-site Coulomb interactions of generalized orbital states, such as molecular orbitals (MOs) and Kohn-Sham states. For example, the band gap of 1T-TaS_2_ can be corrected under the DFT+GOU scheme by applying a U potential on the charge density wave state^25,39^. These studies reveal that advanced theoretical approaches enable a precise investigation of correlated condensed matter systems.

Here, we investigate the insulating nature and metal-insulator transition in the *κ*-ET-CN system through extensive DFT calculations. The *κ*-ET family exhibits discrepancies between experimental observations and DFT calculations, particularly in their temperature- and pressure-dependent phase diagrams^1,17^. While experimental observation demonstrates that *κ*-ET-CN is insulating under ambient conditions, for instance, DFT calculations describe a metallic band structure. This inconsistency hinders the understanding of intriguing phenomena such as metal-insulator transitions and light-induced superconductivity^2,11,12^. To address this problem, we analyze the band structure starting from the constituent BEDT-TTF dimer’s MO energy levels, which are the basis units of the *κ*-ET-CN lattice. We verify that the highest occupied MO and the lowest unoccupied MO (HOMO-LUMO) gap of the BEDT-TTF dimer, corrected by the DFT+GOU method that applies the Hubbard U potential on molecular orbital states rather than atomic orbitals, provides an insulating electronic structure of *κ*-ET-CN and properties consistent with experimental observations. Additionally, we qualitatively reproduce the superconducting dome’s dependence on the external pressure, starting from the insulating electronic structure at ambient conditions. We derive a new set of TB parameters that can be used to accurately investigate quantum spin liquids and light-enhanced superconductivity.

## Results

### Electronic structure of a molecular solid from molecular orbital states

**Fig. 1:**
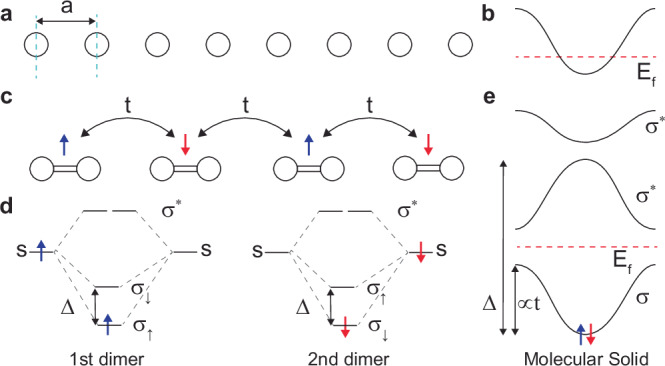
Electronic structure of quarter-filled 1D hydrogen chain. **a** Geometry of a one-dimensional atomic crystal with lattice parameter *a*. **b** Metallic band structure of the one-dimensional crystal, for equal atomic spacing and at quarter-filling. **c** One-dimensional crystal with periodicity 2*a*, formed by dimerization, at quarter-filling. **d** Schematic of the orbital energy levels of a dimer molecule with HOMO-LUMO gap Δ. The lowest levels *σ*_*↑*_ and *σ*_*↓*_ are spin polarized and singly degenerate. **e** Insulating band structure of the one-dimensional molecular crystal driven by dimerization.

Before discussing the main findings of this work for *κ*-ET-CN, we first introduce the most straightforward model to simplify the electronic structure of molecular solids. A metallic band structure can be constructed by a one-dimensional chain of equally spaced hydrogen atoms at quarter-filling (see Fig. 1a, b). By distorting this configuration, orbital hybridization between adjacent molecules (see Fig. 1c) can open a band gap by dimerization, analogous to the Peierls distortion. To describe this scenario, we introduce a molecular HOMO-LUMO gap (Δ) between the spin degenerate bonding orbitals (*σ*) of each dimer, which depends on the dimer occupation as depicted in Fig. 1d. When the dimers form a solid, the competition between inter-site hopping (*t*) and Δ determines the final bulk band structure and energy gap (see Fig. 1e), and generally favors an antiferromagnetic alignment. For example, a large HOMO-LUMO gap (Δ) can result in an insulating band structure, while a small gap leads to a metallic band structure. This example demonstrates that the basis molecule’s MO levels can be crucial in determining the insulating electronic structure of molecular solids.

**Fig. 2:**
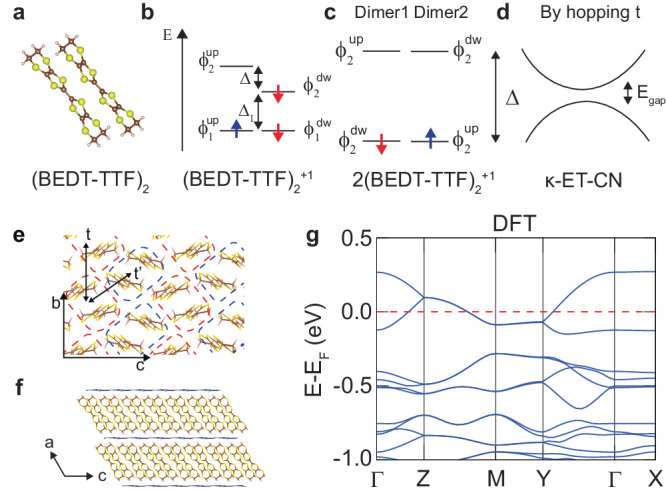
Atomic and electronic structures of *κ*-ET-CN from BEDT-TTF dimers. **a** Atomic geometry of BEDT-TTF dimer. The schematic diagram for a molecular orbital energy level of (**b**) a charged dimer (BEDT-TTF)_2_^+1^, (**c**) charged two-dimer 2(BEDT-TTF)_2_^+1^, and (**d**) band structure of a molecular solid constructed by 2(BEDT-TTF)_2_^+1^. Atomic geometry of *κ*-ET-CN in (**e**) a–c and (**f**) b–c planes. **g** Band structure of *κ*-ET-CN evaluated by DFT (PBE) functional. In (**e**), the dashed blue and red boxes indicate the two-dimer basis for *κ*-ET-CN, and bold black double-sided arrows indicate the hopping between dimers for the nearest (*t*) and the next nearest (${t}^{{\prime} }$) neighboring dimer.

In the rhombohedral lattice, *κ*-ET-CN consists of two BEDT-TTF molecular dimers forming a quasi-2D layer and a Cu_2_(CN)_3_ metal frame layer in the space group *P*2_1_/*c* (see Fig. 2a). A quasi-2D triangular lattice is constructed using two dimers (2(BEDT-TTF)_2_) in the *b* − *c* plane, as shown in Fig. 2e. Because of the charge transfer between 2(BEDT-TTF)_2_ dimers and Cu_2_(CN)_3_ layers, each (BEDT-TTF)_2_ dimer becomes +1 cation and Cu_2_(CN)_3_ layer is -2 anion in *κ*-ET-CN system. Before investigating the electronic structure of *κ*-ET-CN, we first focus on the MO levels in (BEDT-TTF)_2_^+1^ (dimer) and 2(BEDT-TTF)_2_^+1^ (two-dimer) with Perdew–Burke–Ernzerhof (PBE) functional and van der Waals interaction (D3)^40,41^. In the dimer, 0.14 eV HOMO-LUMO gap (Δ) is evaluated with a spin doublet configuration by an odd number of electrons (see Fig. 2b and Table 1). In the two-dimer case, inter-dimer hybridization leads to a smaller HOMO-LUMO gap (Δ = 0.11 eV), as shown in Fig. 2c and Table 1. We expect that the band structure of *κ*-ET-CN is determined by a band dispersion of the HOMO and LUMO states in the two-dimer as a quarter-filled 1D chain case (see Fig. 2d). The metallic band structure of *κ*-ET-CN, as previously reported in a theoretical study^17^, is achieved with 0.11 eV HOMO-LUMO gap of two-dimer (see Fig. 2g) using PBE+D3 functional.

**Table 1:** HOMO-LUMO gaps (Δ) of dimer (BEDT-TTF)_2_^+1^ and two-dimer 2(BEDT-TTF)_2_^+1^ with various functionals

Δ (eV)	PBE+D3	PW6B95+D4	HF
dimer	0.14	1.3	2.96
two-dimer	0.11	0.66	2.39

### Electronic structure dependence of the HOMO-LUMO gap of a dimer in *κ*-ET-CN

**Fig. 3:**
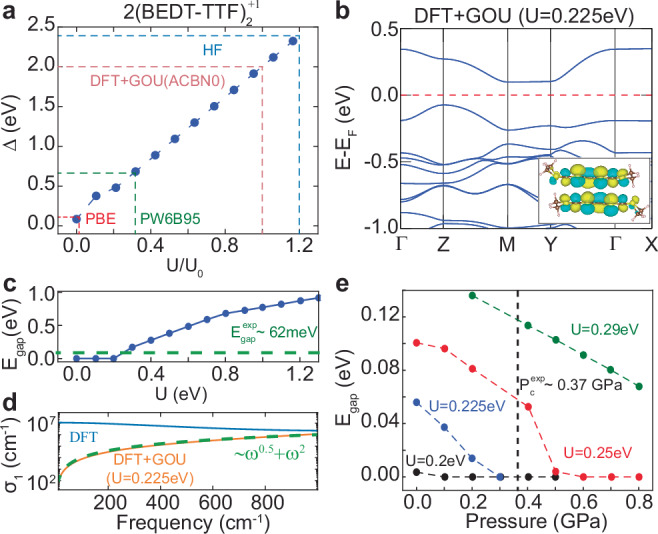
Insulating band structure of *κ*-ET-CN constructed by enough HOMO-LUMO gap of a dimer and reproduced properties. **a** The HOMO-LUMO gap of the charged two-dimer computed by various functionals and DFT+GOU with various U parameters. **b** Band structure of *κ*-ET-CN evaluated by DFT+GOU method with *U* = 0.225 eV. **c** Band gap of *κ*-ET-CN calculated by various U parameters for DFT+GOU. **d** Optical conductivity *σ*_1_ of the *κ*-ET-CN evaluated by DFT and DFT+GOU(*U* = 0.225 eV). **e** U parameters- and pressure-dependency on the band gap of *κ*-ET-CN. The inset of (**b**) indicates the *ϕ*_2_ state of the dimer system, which consists of the valence band maximum and conduction band minimum states of *κ*-ET-CN.

To understand the electronic structure of *κ*-ET-CN and its dependence on the HOMO-LUMO gap of the dimer, we evaluate the two-dimer HOMO-LUMO gap with electronic structure methods of different complexity and accuracy, including PBE, Hartree-Fock (HF), PBE+GOU, and hybrid functionals (see Fig. 3a). The PBE functional predicts the lowest and HF the highest HOMO-LUMO gap for the charged two-dimer system (Δ^PBE^ = 0.11 eV and Δ^HF^ = 2.4 eV). This difference originates from the underestimation and overestimation of on-site Coulomb interactions with the PBE functional and HF calculation, respectively^29^. In addition, DFT+GOU with Agapito-Curtarolo-Buongiorno Nardelli functional (ACBN0)^25,34,35^, which determines the U value self-consistently for MO of the dimers as ${U}_{{\rm{ACBN0}}}^{{\rm{GOU}}}=2.1$ eV in vacuum, provides a large HOMO-LUMO gap (${\Delta }_{{\rm{ACBN0}}}^{{\rm{GOU}}}=2.0$ eV). Considering the ACBN0 method is based on the HF-type electron-electron interaction, it is reasonable that the corresponding DFT+GOU(ACBN0) provides a similar result as HF calculation^42,43^. On the other hand, the hybrid functional (PW6B95), which is expected to provide the most accurate MO levels among the functionals we used, introduces an intermediate HOMO-LUMO gaps of Δ^PW6B95^ = 0.66eV. This result indicates that the Δ of the two-dimer significantly depends on the DFT functionals. By varying the U parameters for DFT+GOU, on the other hand, the HOMO-LUMO gap for various functionals can be reproduced, as shown in Fig. 3(a). In Supplementary Section 1 and Fig. S1, we discuss the electronic structure evaluated using DFT+GOU, DFT+U, and the constrained random-phase approximation under various conditions^44^. Our results show that DFT+GOU successfully corrects the HOMO-LUMO gap as intended by the inclusion of the Hubbard U potential, whereas DFT+U fails to achieve this.

The HOMO-LUMO gap of the two-dimer leads to an insulating band structure for *κ*-ET-CN. The hybrid functional, known for aligning theoretical energy gaps with experimental observations, is expected to provide an accurate electronic structure in this system^32,45^. However, the computational cost is prohibitively high for the *κ*-ET-CN. As a practical alternative, we use the DFT+GOU method to evaluate the electronic structure of *κ*-ET-CN with the corrected HOMO-LUMO gap. With a specific *U* value (*U* = 0.225 eV), the insulating band structure with an antiferromagnetic ordering configuration and a magnetic moment of ± 1*μ*_*B*_ for each dimer site is achieved by the DFT+GOU method (see Fig. 3b). This *U* value is estimated by the ratio of *U* value between ACBN0 (${U}_{{\rm{ACBN0}}}^{{\rm{GOU,vac}}}=2.1$ eV) and the hybrid function (*U*^PW6B95,vac^ = 0.69 eV) in vacuum as follows: $U \sim 0.24{U}_{{\rm{ACBN0}}}^{{\rm{GOU,k-salt}}}=0.225$ eV, when ${U}_{{\rm{ACBN0}}}^{{\rm{GOU,k-salt}}}=0.94$ eV. It indicates that the enough HOMO-LUMO gap of the dimer opens the band gap of *κ*-ET-CN. When we scan the band gap of *κ*-ET-CN by increasing *U* values for the dimer’s MO states, it is found that a larger HOMO-LUMO gap (Δ) leads to an increased band gap (*E*_gap_). On the other hand, a smaller Δ achieved by a lower *U* (< 0.2 eV) value closes the band gap due to band dispersion (see Fig. 3c). These results indicate that the band gap of *κ*-ET-CN strongly relies on the HOMO-LUMO gap of dimers, which is modulated by the *U* value for the dimer’s MO states. A detailed discussion about the determination of *U* values is provided in the Supplementary Section 2.

### Comparison between experimental observations and DFT+GOU calculations

The electronic structure of *κ*-ET-CN described by DFT+GOU with *U* = 0.225 eV provides consistent material properties aligning with experimental observations^46^. First, the band gap (*E*_gap_ = 56 meV) evaluated by DFT+GOU (*U* = 0.225 eV) and experimental measurement provides ${E}_{{\rm{gap}}}^{\exp }=62$ meV (see Fig. 3c)^46^. Second, the optical conductivity (*σ*_1_(*ω*)) in the *κ*-ET-CN also shows *ω*^0.5^ and *ω*^2^ behavior (see Fig. 3d)^46^. Due to the metallic band structure from the DFT functional, its optical conductivity decreases with increasing light frequency. Third, the pressure-induced metal-insulator transition is achieved with DFT+GOU (*U* = 0.225 eV) at ${P}_{c}^{{\rm{GOU}}} \sim 0.3$ GPa and it is observed experimentally at ${P}_{c}^{\exp } \sim 0.37$ GPa^1^. The band gap of *κ*-ET-CN, evaluated by varying pressures and U values for DFT+GOU (Fig. 3e), shows that higher U values increase the band gap at *P* = 0 GPa and raise the critical pressure for the metal-insulator transition. These results indicate that the electronic structure of dimers provides consistent properties of *κ*-ET-CN, as observed in experiments.

**Fig. 4:**
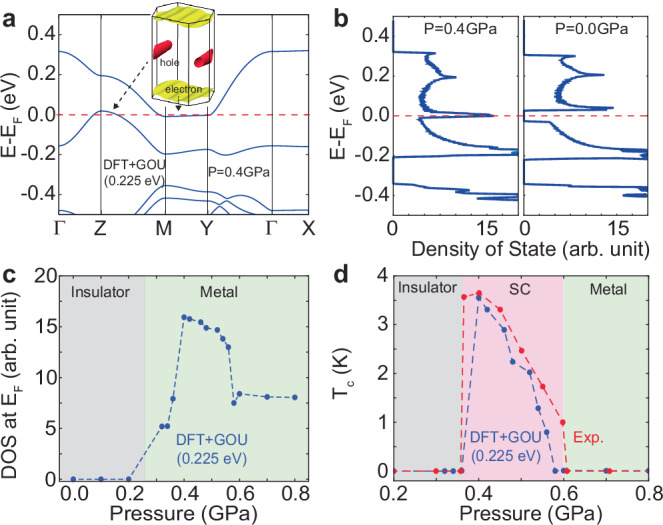
Pressure-induced metal-insulator transition and origin of superconducting dome. **a** Band structure of *κ*-ET-CN with 0.4 GPa pressure using DFT+GOU (*U* = 0.225 eV). **b** Density of state of *κ*-ET-CN with (left) 0.4 GPa, and (right) 0.0 GPa pressures evaluated by the DFT+GOU (*U* = 0.225 eV). **c** Density of state at Fermi level with varying pressure using DFT+GOU (*U* = 0.225 eV). **d** Estimated critical temperature by BCS equation using the density of state at the Fermi level evaluated by DFT+GOU (*U* = 0.225 eV) and observed experimental data^1^ The inset of (**a**) indicates the Fermi surface of *κ*-ET-CN with 0.4 GPa pressure using DFT+GOU (*U* = 0.225 eV).

Having discussed the electronic structure of *κ*-ET-CN in its insulating phase and across the metal-insulator transition, we now turn to the superconducting dome induced by external pressure, analyzing it within the framework of BCS theory^1,47,48^. In the experiment, a hydrostatic pressure of 0.37 GPa not only leads to the metal-insulator transition but also provides the highest T_*c*_ value of the superconducting dome^1^. To understand this behavior, we investigate the electronic structure of *κ*-ET-CN with external pressure with *U* = 0.225 eV for DFT+GOU. The *κ*-ET-CN with 0.37 GPa external pressure has a flat electronic band along the M-Y line at the Fermi level, as shown in Fig. 4a. This flat band induces a sharp peak in the density of states (DOS) at the Fermi level, which is unoccupied at zero pressure but becomes aligned near the Fermi level under external pressure (see Fig. 4b). Under higher external pressure, the DOS at the Fermi level (*N*_*F*_) is reduced, due to the downward shift of the flat band falling below the Fermi level (see Fig. 4c). Although the *κ*-ET family is expected to exhibit non-conventional superconductivity mediated by spin fluctuations^49^, here we naively estimate the critical superconducting temperature (*T*_*c*_) using the BCS equation based on the DOS profile at the Fermi level,^50^: ${k}_{B}{T}_{c}=1.134\,{E}_{D}\,\exp (-1/({N}_{F}V))$, where *E*_*D*_ and *V* are the Debye frequency and the effective electron-electron coupling potential, respectively. We set the Debye frequency *E*_*D*_ = 0.2 eV to the C-C stretching vibration frequency of the BEDT-TTF molecule, which has a strong electron-phonon coupling and is expected to contribute to the formation of electron-electron pairing^4^, and use *N*_*F*_ from the DFT+GOU calculation with *U* = 0.225 eV. Because other molecular solid compounds (alkali-doped C_60_) and other unconventional superconductors (rhombohedral trilayer graphene) exhibit a dependence of the superconducting transition temperature (*T*_*c*_) on the DOS at the Fermi level^5,19,51–53^, we assume that the *κ*-family follows a similar trend. By fitting the parameter *V*, we find that *V* = 9.5 × 10^−3^ DOS^−1^ yields *T*_*c*_ values that are consistent with experimental observations across a wide range of external pressures (see Fig. 4d)^1^. Unlike our estimation here, additional force and electron-phonon coupling calculations within the DFT+GOU framework are required for an accurate first-principles-based estimation, following the procedures established in previous developments^54–56^. Although this simplified approach neglects several critical factors, such as pressure-dependent electron-phonon coupling, Debye frequency variations, *U* values, and other unknown contributions, our estimate nonetheless qualitatively reproduces the characteristic superconducting dome, in good agreement with experimental data.

### Parameterization of the tight-binding model Hamiltonian

Model Hamiltonians have been employed to investigate the fascinating many-body physics of the *κ*-ET-X family^7,10,13–16,57^. For example, the temperature-pressure phase diagram has been theoretically calculated using the half-filled one-band Hubbard model, and spin ordering has been studied using Heisenberg-like spin Hamiltonians^57^. Although Hubbard model studies report a metal-insulator transition driven by the competition between U and t^16,58,59^, DFT-based parameters do not reproduce the pressure-dependent metal-insulator transition observed experimentally^17^. Here, we provide model parameters for a Hamiltonian derived from the dimer MO levels of *κ*-ET-CN, in order to reproduce its electronic structure. For the simplest TB model, we employ the HOMO and LUMO states (${\phi }_{2}^{\sigma }$) of the dimers as the local orbital basis (see Fig. 2c). The anisotropic triangular lattice Hubbard Hamiltonian used to fit the band structure is given by: $H=t{\sum }_{\langle ij\rangle ,\sigma }({c}_{i,\sigma }^{\dagger }{c}_{j,\sigma }+H.c.)+{t}^{{\prime} }{\sum }_{[ij],\sigma }({c}_{i,\sigma }^{\dagger }{c}_{j,\sigma }+H.c.)+\Delta {\sum }_{i,\sigma }(1-{n}_{i,\sigma }/2)$, where 〈*ij*〉 and [*ij*] are summations over nearest neighbors on equivalent and inequivalent sites, respectively. The hopping interactions between MO states, described by the *t* term for equivalent sites and the ${t}^{{\prime} }$ term for inequivalent sites, are employed to describe the band dispersion (see Fig. 2e). For the antiferromagnetic solution, we set the site occupations (see Fig. 2c) as follows: *n*_1,*↓*_ = *n*_2,*↑*_ = 1 and *n*_1,*↑*_ = *n*_2,*↓*_ = 0. The metallic band structure from DFT calculation provides *t* = 50 meV and ${t}^{{\prime} }=39$ meV (${t}^{{\prime} }/t=0.78$) without the local HOMO-LUMO gap (Δ = 0) of the dimer, which is consistent with previous reports (${t}^{{\prime} }/t=0.83$)^2,17^. On the other hand, the insulating band structure of the DFT+GOU (*U* = 0.225 eV) calculation provides *t* = 52 meV, ${t}^{{\prime} }=39$ meV, and Δ = 239 meV (${t}^{{\prime} }/t=0.75$ and Δ/*t* = 4.59). The parameters extracted from DFT and DFT+GOU indicate that GOU terms mainly modify the HOMO-LUMO gap of the two-dimer, rather than the hopping terms. The band structure at 0.4 GPa, which results from the metal-insulator transition, provides the following parameters: *t* = 56 meV, ${t}^{{\prime} }=45$ meV, and Δ = 192 meV (${t}^{{\prime} }/t=0.80$ and Δ/*t* = 3.43). It indicates that external pressure reduces the two-dimer’s HOMO-LUMO gap (Δ/*t*), and increases inter-dimer hopping amplitudes (*t* and ${t}^{{\prime} }/t$), which both act to close the band gap of *κ*-ET-CN. Notably, previous models only consider the gap (Δ_1_) between HOMO and HOMO-1 of dimer (see Fig. 2b), by estimating it from the hopping between nearest monomers (Δ_1_ ~ 2*t*_1_ in Fig. 2b), not the HOMO-LUMO gap of the two-dimer (Δ)^2,17^. Our results indicate that the two-molecule dimer unit of the HOMO-LUMO gap is essential to realize the insulating electronic structure. We suggest that these parameters can be exploited for follow-up theoretical many-body studies addressing, for example, the spin liquid phase, light-enhanced superconductivity, and quantum criticality in *κ*-ET-X family^4,7,11,13,14^. In the Supplementary Section 3 and 4, we discuss details of the different TB lattice models developed and band structure under the various pressure (See Figs. S2, S3, S4, and S5)^60^.

## Discussion

We have demonstrated that the insulating phase and metal-insulator transition in *κ*-ET-CN is controlled by the HOMO-LUMO gap of the dimer, which constitutes the fundamental periodic unit in this organic molecular solid. From the MO level of (BEDT-TTF)_2_^−1^, we found that the hybrid functional provides the most realistic HOMO-LUMO gap for (BEDT-TTF)_2_^−1^ among various DFT functionals, but can be reproduced by the DFT+GOU scheme using an appropriate *U* value. When we extend this analysis to bulk *κ*-ET-CN, the electronic structure of *κ*-ET-CN obtained from DFT+GOU (*U* = 0.225 eV) calculations reveal experimentally consistent behaviors: a band gap of magnitude *E*_gap_ = 53 meV, an optical conductivity scaling as ~ *ω*^0.5^ + *ω*^2.0^, and a critical pressure for metal-insulator transition of *P*_*c*_ = 0.3 GPa. Additionally, the pressure-induced superconducting dome is roughly reproduced compared with experimental observations. These results indicate that an accurate evaluation of the internal structure of the molecular dimers, which constitute the basic unit of the molecular solid, are required for accurate description of the electronic structure of bulk *κ*-ET-CN. When this is achieved, first-principles calculations provide material properties consistent with experimental observations. Additionally, our derived TB parameters, which take into account the corrected HOMO-LUMO gap of the two-dimer, can be utilized for further many-body studies. We also provided a modified lattice model description of this molecular solid, complementing the widely used approach in the literature by adding the effect of the molecular dimer gap^2,17^. This new TB Hamiltonian parameterization will stimulate further work to address the competition between local and long-range correlation effects in these molecular solids, such as light-enhanced superconductivity and spin liquid phases. Our study provides microscopic insights into the electronic structure of molecular solids, and opens an avenue for a theoretical analysis of the exotic behaviors in the *κ*-ET family^4,7,11,13,14^.

## Methods

### Computational details

We performed DFT calculations using the Quantum Espresso package^61^. The wavefunction is described using the projector-augmented-wave method using pslibrary 1.0.0 version^62^ and the plane wave basis set with an 80 Ry energy cut-off. We employed the PBE-type exchange-correlation functional, hybrid functional, and DFT+GOU methods to describe the electron-electron exchange and correlations^21,25,40^. The on-site Coulomb interaction for 8 states below the HOMO state and four states above the LUMO level is corrected by the DFT+GOU method. The atomic geometry is fully relaxed with PBE plus van der Waals D3 (PBE+D3) functional^41^. The calculated lattice constants for *κ*-ET-CN are *a* = 15.8 Å, *b* = 8.46 Å, *c* = 13.7 Å, and *β* = 110. 8^∘^ in the *P*2_1_/*c* space group under the ambient pressure. The Brillouin zone is sampled with 4 × 4 × 4 **k**-point mesh for the molecular solid system. A large cubic lattice (*a* = 30.0 Å) with three-dimensional periodic boundary conditions is employed for the molecular calculations. To achieve the pressured geometry of *κ*-ET-CN, we proceeded with lattice and ionic position relaxation with a given hydrostatic pressure along the a, b, and c axes using the PBE+D3 functional. The lattice relaxation is achieved with a given force criterion (∣*F*∣ < 10^−5^*Ry*/*bohr*) for each atom and a given stress criterion (∣*P*∣ < 0.005 GPa).

The estimation of the effective Hubbard U for the *C* − *p* and *S* − *p* orbitals was done using the Octopus code^63^, using the ACBN0 functional method^34,64^. We employed here a grid spacing of 0.3 Bohr, a box made of the union of a sphere around each atom of the dimer, with a radius of 10 Bohr for each sphere, and pseudo-dojo PBE pseudopotentials^65^. We employed the same relaxed geometry as in the rest of the simulations.

Molecular calculations on the BEDT-TTF dimer and two dimers were also performed using the Orca quantum chemistry software^66^. The HOMO-LUMO gaps of both dimer and two dimers were calculated with the HF method, as well as with the DFT method by employing the generalized gradient approximation PBE exchange-correlation functional^40^, and the hybrid meta-generalized gradient approximation PW6B95 exchange-correlation functional with the def2-QZVPP^67,68^. We also double-checked our calculation with cc-pVDZ, and def2-SVP basis set^69,70^. Moreover, the DFT calculations with the PBE and PW6B95 functionals utilize the D3^41^ and D4^71^ dispersion corrections, respectively.

### Molecular orbital basis set for DFT+GOU calculation

In the DFT+GOU formalism, the correction of on-site Coulomb interaction is applied within a subspace defined by selected molecular orbital states. Unlike the DFT+U method, which targets the atomic orbital states, the DFT+GOU approach corrects the energy level of molecular orbital states. For example, the conventional DFT+U method counts the occupation of *p*- or *d*-orbitals in Kohn-Sham states to construct the Hubbard U potential, while DFT+GOU evaluates the occupation of generalized orbitals in Kohn-Sham states using generalized projectors, such as HOMO and LUMO states and Wannier functions^25^. Notably, Hubbard U potential shifts the energy level of target states depending on their occupations^33^. For the BEDT-TTF dimer, we can select the highest occupied molecular orbital (HOMO) and the lowest unoccupied molecular orbital (LUMO) as the subspace for correction. We examined the band structure with different configurations to determine the optimal subspace size. Since the valence band maximum (VBM) and conduction band minimum (CBM) are well-separated from other bands, using only the HOMO and LUMO states as projectors for Hubbard potentials, is sufficient to correct the on-site Coulomb interaction and open the band gap (see Supplementary Section S5 and Fig. S6 for details). We also compared this with a more extensive configuration using a 16-basis set (8 occupied and 8 unoccupied states). We found that the minimum and extended configurations provide a consistent electronic structure near the Fermi level.

## Supplementary information

Supplementary information

## Data Availability

The data that support the findings of this article are openly available at 10.6084/m9.figshare.28245266.

## Code availability

In-house modification for DFT+GOU used in this study is available from the corresponding authors upon request.
