# Supplementary material for: Origin of the insulating phase and metal-insulator transition in the organic molecular solid κ-(BEDT-TTF)2Cu2(CN)3
Source: NPJ Comput Mater. 2026 Jan 17;12(1):93. doi: 10.1038/s41524-026-01960-y (PMC12913020; doi:10.1038/s41524-026-01960-y)
Supplement: Supplementary file 1 — Supplementary information [file 41524_2026_1960_MOESM1_ESM.pdf]

Supplementary Information: Origin of the insulating phase and metal-insulator transition in the organic molecular solid κ-(BEDT-TTF)$_2$Cu$_2$(CN)$_3$

Dongbin Shin,[1,2,*] Fabijan Pavošević,[3] Nicolas Tancogne-Dejean,[2]
Michele Buzzi,[2] Emil Viñas Boström,[2] and Angel Rubio[2,4,5,†]

[1]*Department of Physics and Photon Science,*
Gwangju Institute of Science and Technology (GIST), Gwangju 61005, Republic of Korea

[2]*Max Planck Institute for the Structure and Dynamics of Matter and*
Center for Free Electron Laser Science, 22761 Hamburg, Germany

[3]*Algorithmiq Ltd., Kanavakatu 3C, FI-00160 Helsinki, Finland*

[4]*Nano-Bio Spectroscopy Group, Departamento de Fisica de Materiales,*
Universidad del País Vasco UPV/EHU- 20018 San Sebastián, Spain

[5]*Center for Computational Quantum Physics (CCQ),*
The Flatiron Institute, 162 Fifth avenue, New York NY 10010.

SUPPLEMENTARY SECTION S1: EFFECT OF HUBBARD U WITH ATOMIC ORBITAL PROJECTORS IN CONVENTIONAL DFT+U

A previous study shows that DFT+U fails to correct the on-site Coulomb interaction in MOs or general hybridized states using atomic orbital projectors [1]. Here, we verify that applying Hubbard U potential on C-p orbitals and S-p orbitals under the DFT+U scheme doesn't modify the HOMO-LUMO gap of the two-dimer system ($\Delta^{DFT+U} \sim 0.11$eV) effectively in the wide range of U values ($0 \leq U \leq 10$ eV). We first evaluate the self-consistently evaluated U value for each atomic site using the ACNB0 method in the dimer system [2]. We found $U^{C}_{ACBN0} = 3.8$ eV for carbon atoms and $U^{S}_{ACBN0} \sim 1.7$ eV for sulfur atoms in the dimer system. Similar to previous reports [1], Hubbard U potential with atomic orbital projectors does not intentionally shift the energy level of the generalized (or molecular) state.

We also verified that this HOMO-LUMO gap in the dimer is robust with a wide range of U ($0 < U < 10$ eV) for atomic orbital states. For example, we assign a single value of U to all C and S atoms, as determined by ACBN0. From the DFT and DFT+U calculations, we achieved an electronic structure without a band gap (see Fig. S1). However, the overall bands shift downward, except for the metal-frame states highlighted by arrows in Fig. S1c[3]. Since the Hubbard potential is applied only to the atomic orbital states of each atom consisting of the BEDT-TTF molecules under the DFT+U scheme, this shift suggests that the molecular orbital states are uniformly affected by the Hubbard potential, irrespective of their occupations. This behavior highlights the crucial distinction between DFT+U with an atomic orbital basis set and DFT+GOU, as previously reported [1]. Notably, Hubbard U potential in DFT is constructed to shift down and up for occupied and unoccupied bands, respectively [4]. Unlike DFT+GOU, DFT+U with an atomic orbital basis set fails to count the occupations of molecular orbitals. Consequently, occupied and unoccupied ϕ_2 states are not shifted downward and upward, respectively. Furthermore, we observed that the band structure remains unchanged under the external pressure in DFT+U calculations. As shown in Fig. S1d, a similar band structure is found in 0.7 GPa, calculated using DFT+U, as it is without pressure. These results indicate that DFT+U with an atomic orbital basis set does not modify the dimer's HOMO-LUMO gap, resulting in a purely metallic band structure.

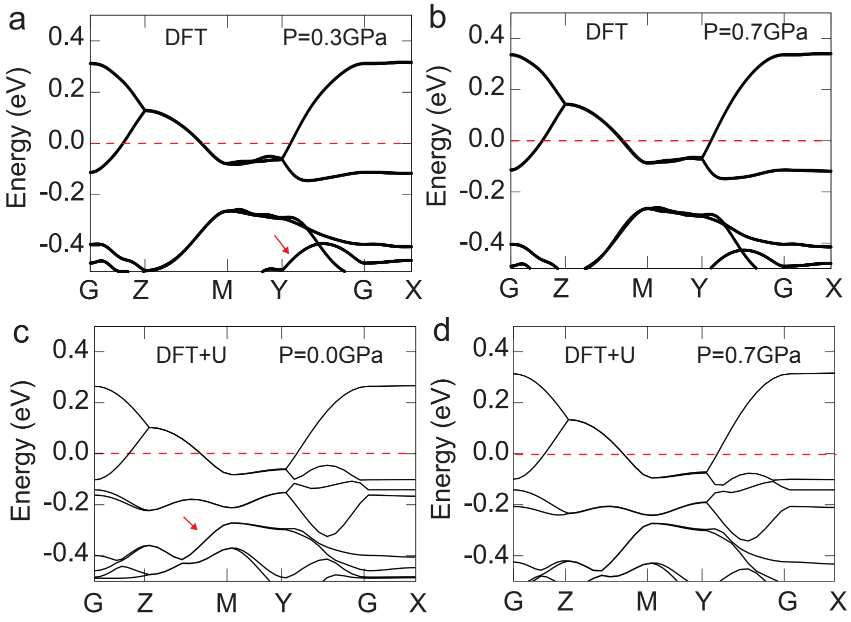

FIG. S1: **a-b** DFT band structure for κ-ET-CN with (**a**) 0.3 GPa and (**b**) 0.7 Gpa. **c-d** DFT+U band structure for κ-ET-CN with (**c**) 0.0 GPa and (**d**) 0.7 Gpa. In the insets of (**a**) and (**c**), the red arrows indicate the metal-frame bands.

SUPPLEMENTARY SECTION S2: DETERMINING U VALUE FOR DFT+GOU CALCULATION

To determine the Hubbard U of the dimer, we performed DFT calculations with various functionals in the main text. The ACBN0 is employed to evaluate the Hubbard U value self-consistently from a reduced subspace for HF-type Coulomb interaction [5]. On the other hand, our results show that the ACBN0 provides too large a HOMO-LUMO gap for a dimer compared with its value computed by the hybrid functional. Therefore, we roughly estimate the appropriate U value from the HOMO-LUMO gap evaluated by the hybrid functional. For example, the DFT+GOU with $U \sim 0.3U^{\mathrm{GOU}}_{\mathrm{ACBN0}}$ provides a similar HOMO-LUMO gap as a hybrid functional calculation, as shown in Fig. 3a of the main text. Among various U value, we found that the DFT+GOU with an appropriate U value ($U \sim 0.24U^{\mathrm{GOU}}_{\mathrm{ACBN0}} =$ 0.225 eV) provides consistent material properties as experimental observations. Notably, this ratio in κ-ET-CN is comparable with the ratio between ACBN0 and PW6B95 for a Δ of the two-dimer in vacuum ($U \sim 0.3U^{\mathrm{GOU}}_{\mathrm{ACBN0}} = 0.63$ eV). It indicates that DFT+GOU with $U = 0.225$ eV yields a similar correction to the energy level of the dimer as the hybrid functional and provides a realistic electronic structure of κ-ET-CN.

SUPPLEMENTARY SECTION S3: FITTING FOR THE TIGHT-BINDING MODEL HAMILTONIAN

To describe the two bands near the Fermi level in the κ-ET-CN, we considered the two-site periodic tight-binding Hamiltonian for a trigonal lattice as described in Fig. 2e of the main text, which is given as follows:

$$H = \sum_{<ij>,\sigma} t(c^{\dagger}_{i,\sigma}c_{j,\sigma} + H.c.) + \sum_{[ij],\sigma} t'(c^{\dagger}_{i,\sigma}c_{j,\sigma} + H.c.) + \Delta \sum_{i,\sigma}(1 - 0.5n_{i,\sigma}) \quad (1)$$

, when $< ij >$ and $[ij]$ are summations for the same sites in the nearest neighbor lattice and nearest neighboring different sites, respectively.

To fix the antiferromagnetic solution, we set the occupation for each spin state and site as follows:

$$< \hat{n}_{1,\downarrow} >= 1,\ < \hat{n}_{2,\uparrow} >= 1, \text{and}\ < \hat{n}_{1,\uparrow} >=< \hat{n}_{2,\downarrow} >= 0, \quad (2)$$

they indicate a single spin-up occupied state for 1st dimer and a single spin-down occupied state for 2nd dimer. Notably, there are two sites ($i = 1$ and $i = 2$) in the primitive trigonal lattice. These configurations lead to the matrix elements for on-site energy terms as follows:

$$\begin{aligned} < 1,\uparrow |\hat{H}_{on-site}|1,\uparrow>&= \Delta/2, \\ < 1,\downarrow |\hat{H}_{on-site}|1,\downarrow>&= -\Delta/2, \\ < 2,\uparrow |\hat{H}_{on-site}|2,\uparrow>&= -\Delta/2, \\ < 2,\downarrow |\hat{H}_{on-site}|2,\downarrow>&= \Delta/2. \end{aligned}$$

With this assumption, we used the pythTB code to extract the parameters for the tight-binding model Hamiltonian [6]. From the DFT band structure, $t = 50$ meV, $t' = 39$ meV, and $\Delta = 0.0$ meV are achieved for the tight-binding parameters. We found that tight-binding band structures reproduced the DFT band structure, as shown in Fig. S2a. When the band gap of κ-ET-CN is opened by the DFT+GOU method, $t = 52$ meV, $t' = 39$ meV, and U value $\Delta = 239$ meV are achieved for tight-binding parameters, and this tight-binding band structure accurately reproduces the DFT+GOU band structure, as shown in Fig. S2b. The following parameters are achieved under the 0.4 GPa external pressure: $t = 56$ meV, $t' = 45$ meV, and $\Delta = 192$ meV (See Fig. S2c). Because there is only a Δ value difference in fitting parameters in these conditions, the band gap is mainly modulated by the pressure dependency of the HOMO-LUMO gap of a dimer.

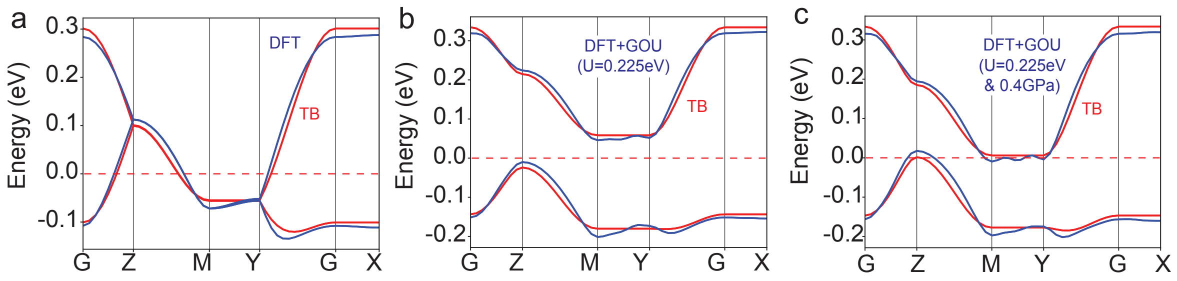

FIG. S2: **a** DFT band structure for κ-ET-CN and its reproduced band structure with tight-binding fitting parameters. **b** DFT+GOU($0.225eV$) band structure for κ-ET-CN and its reproduced band structure with tight-binding fitting parameters. **c** DFT+GOU($0.225eV$) band structure for κ-ET-CN and its reproduced band structure with tight-binding fitting parameters underthe 0.4 GPa external pressure.

We also investigated the tight-binding parameter with other basis sets. For these cases, we include two more valence bands ($\phi_{1,\sigma}$) below the valence band maximum state ($\phi_{2,\sigma}$), as shown in Fig. 2b of the main text. First, we choose the two orbitals (ϕ_1 and ϕ_2) for each dimer site. We employed an extended Hamiltonian for this case as follows:

$$H = \sum_{<ij>,\sigma,l} t_n(c^\dagger_{i,\sigma,l}c_{j,\sigma,l}+H.c.)+\sum_{[ij],\sigma,l} t'_l(c^\dagger_{i,\sigma,l}c_{j,\sigma,l}+H.c.)+\sum_{i,\sigma,l}\Delta_l(1-0.5n_{i,\sigma,l})+\sum_{i,\sigma,l}\epsilon_l n_{i,\sigma,l} \quad (3)$$

where $l = 1, 2$ is for the MO orbital of a dimer state and ϵ_l is on-site energy of l-orbital. We obtain the tight-binding parameters that reproduce the band structure, as shown in Fig. S3. For the electronic structure achieved by DFT calculation, $t_1 = 11.1$ meV, $t_2 = 50.3$ meV, $t'_1 = -41.2$ meV, $t'_2 = 38.9$ meV, $\epsilon_1 = 6.35$ meV, $\epsilon_2 = -385$ meV, $\Delta_1 = 1.2$ meV, and $\Delta_2 = 1.0$ meV are achieved for the tight-binding Hamiltonian. For the electronic structure achieved by DFT+GOU(0.225eV) calculation, $t_1 = 13.5$ meV, $t_2 = 51.8$ meV, $t'_1 = -28.6$ meV, $t'_2 = 39.3$ meV, $\epsilon_1 = 17.1$ meV, $\epsilon_2 = -502$ meV, $\Delta_1 = 59.4$ meV and $\Delta_2 = 238$ meV are achieved for the tight-binding Hamiltonian. Like a previous 1-orbital and 1-site model, the band gap is well modified by Δ_2 for ϕ_2 states.

Second, we construct the tight-binding model for the one-site per BEDT-TTF monomer as in previous studies [7].

$$H = \sum_{(ij)_k,\sigma} t_k(c^\dagger_{i,\sigma}c_{j,\sigma} + H.c.) + \epsilon \quad (4)$$

where $(ij)_k$ and t_k indicate the hopping between specific BEDT-TTF monomers and their parameters as depicted in Fig. S4a. The ϵ indicates the on-site energy for each monomer,

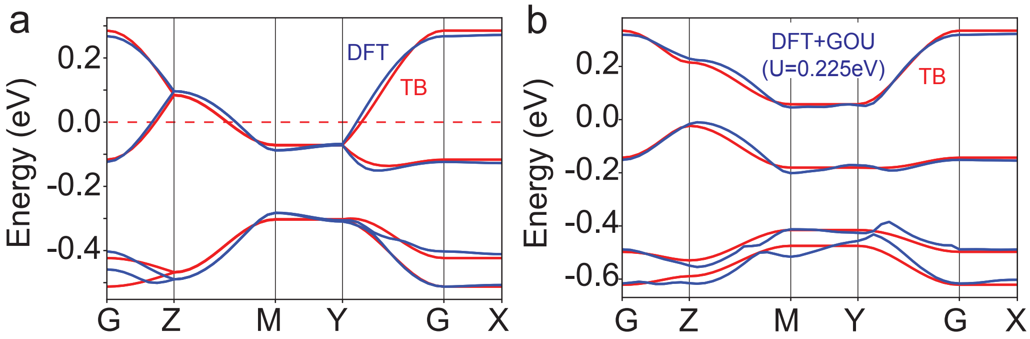

FIG. S3: **a-b** DFT band structure for κ-ET-CN with (**a**) 0.3 GPa and (**b**) 0.7 Gpa. **c-d** DFT+U band structure for κ-ET-CN with (**c**) 0.3 GPa and (**d**) 0.7 Gpa.

so the one value is considered for 4 monomers. For the electronic structure achieved by DFT calculation, $t_1 = 180$ meV, $t_2 = 61.9$ meV, $t_3 = 83.3$ meV, $t_4 = 36.8$ meV, and $\epsilon = -174$ meV are achieved for the tight-binding Hamiltonian. As shown in Fig. S4b, these tight-binding parameters reproduce the DFT result. For the electronic structure achieved by DFT+GOU ($U = 0.225$ eV) calculation, $t_1 = 245$ meV, $t_2 = 64.8$ meV, $t_3 = 72.8$ meV, $t_4 = 50.3$ meV, and $\epsilon = -241$ meV are achieved for the tight-binding Hamiltonian. As shown in Fig. S4c, this tight-binding model Hamiltonian doesn't provide a well-fitted band structure. This discrepancy originates from the lack of terms for the energy gap between $\phi_{2,\uparrow}$ and $\phi_{2,\downarrow}$ of the dimer state from ψ_l of the monomer state. This result indicates that the on-site energy correct for the dimer state (ϕ_2) is required to open the band gap of κ-ET-CN. In previous reports using the constrained random-phase approximation (cRPA), efforts have been made to correct the screened Coulomb interaction in organic solids [8, 9]. However, it was not as effective as our correction for on-site Coulomb interaction with the DFT+GOU method to open the band gap in the κ-salt family. This result indicates that corrections from cRPA might not be as effective.

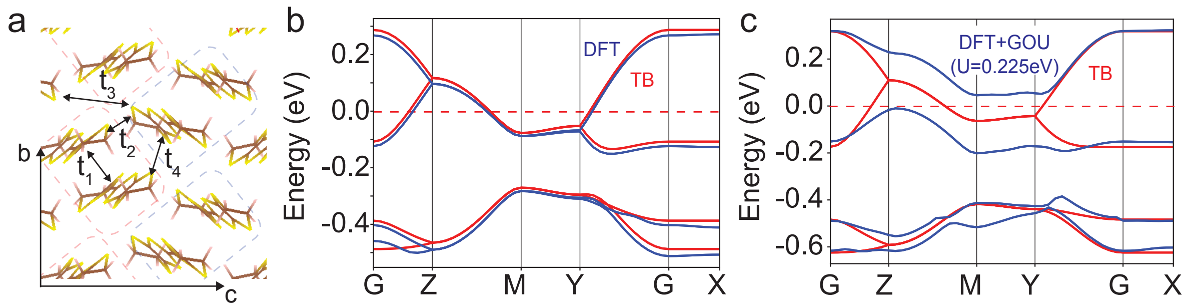

FIG. S4: **a** Schematic image for hopping interactions between the monomers. **b-c** A band structure reproduced using a tight-binding Hamiltonian, with fitting parameters derived from (**b**) DFT and (**c**) DFT+GOU ($U = 0.225$ eV), respectively.

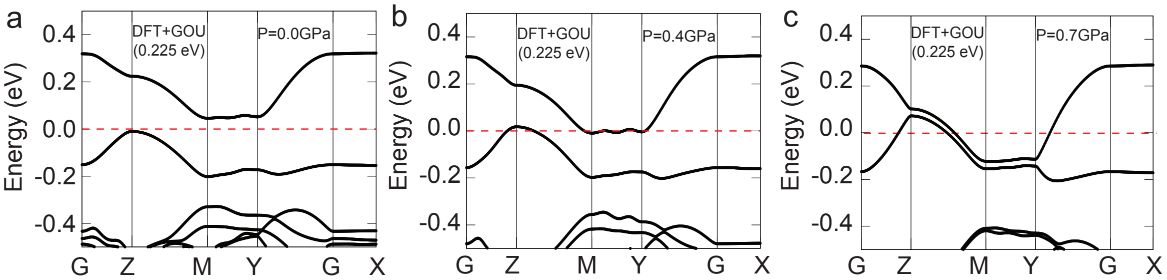

FIG. S5: **a-c** Pressure-dependent band structure (upper) and density of state (below) for (**a**) 0.0 Gpa, (**b**) 0.4 Gpa, and (**c**) 0.7 Gpa evaluated by DFT+GOU($U = 0.225$ eV).

SUPPLEMENTARY SECTION S4: BAND STRUCTURE UNDER THE EXTERNAL PRESSURE

The main text demonstrates that the band gap of κ-ET-CN can be modulated by external pressure, consistent with experimental observations [10]. We found that the band gap gradually closes under external pressure from the initially opened by the corrected HOMO-LUMO gap in the DFT+GOU calculations. Under the DFT+GOU method, κ-ET-CN shows a small band gap without the external pressure, as shown in Fig. S5a. However, applying a pressure of 0.4 GPa closes the band gap, resulting in a peak in the density of states at the Fermi level, as illustrated in Fig. S5b. Under the higher pressure condition (0.7 GPa), the two bands near the Fermi level are significantly closer together, and the flat band shifts below the Fermi level, thereby reducing the density of states (DOS) at the Fermi level. On the other hand, the DFT calculation shows the metallic band structure without the external pressure, as shown in Fig. 2g of the main text. As shown in Figs. S1a and S1b, band structures with 0.3 and 0.7 GPa external pressure, show a resemblance to the DFT band structure without pressure. This result indicates that the electronic structure, without correction of the HOMO-LUMO gap of the dimer, doesn't show significant external pressure dependency.

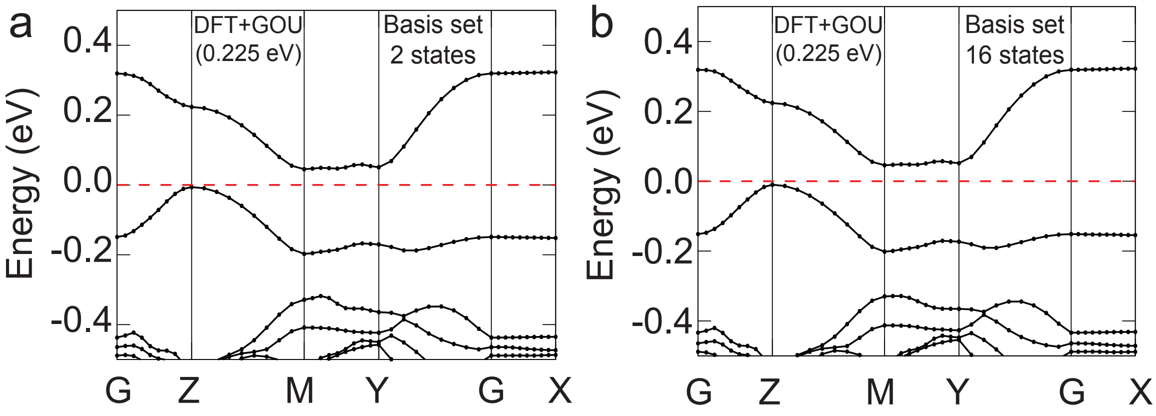

FIG. S6: **a-b** Subspace-dependent band structure with (**a**) 2 states and (**b**) 16 states basis set.

SUPPLEMENTARY SECTION S5: EVALUATING MOLECULAR ORBITAL BASIS SET FOR DFT+GOU CALCULATION

As described in the Method section of the main text, we checked the dependency of the molecular orbital basis set on the band structure. As shown in Fig. S6, the band structures of κ-(BEDT-TTF)$_2$Cu$_2$(CN)$_3$ (κ-ET-CN) with DFT+GOU(0.225 eV) with 2 (HOMO and LUMO) and 16 MO (8 occupied and 8 unoccupied states from the Fermi level) states for GOU projectors are evaluated. These results indicate that minimum configurations (HOMO and LUMO states) are enough to correct the electronic structure of κ-ET-CN. In addition, it shows that the subspace size does not significantly impact the outcome as long as the VBM and CBM states are included in the κ-ET-CN system.

* dshin@gist.ac.kr

† angel.rubio@mpsd.mpg.de

[1] D. Shin, N. Tancogne-Dejean, J. Zhang, M. S. Okyay, A. Rubio, and N. Park, Identification of the mott insulating charge density wave state in 1t-tas2, Phys. Rev. Lett. **126**, 196406 (2021).

[2] N. Tancogne-Dejean, M. J. T. Oliveira, and A. Rubio, Self-consistent DFT+U method for real-space time-dependent density functional theory calculations, Phys. Rev. B **96**, 245133 (2017).

[3] M. Buzzi, D. Nicoletti, S. Fava, G. Jotzu, K. Miyagawa, K. Kanoda, A. Henderson, T. Siegrist, J. A. Schlueter, M.-S. Nam, A. Ardavan, and A. Cavalleri, Phase Diagram for Light-Induced Superconductivity in kappa-$(ET)_2$-X, Phys. Rev. Lett. **127**, 197002 (2021).

[4] V. I. Anisimov, I. V. Solovyev, M. A. Korotin, M. T. Czyyk, and G. A. Sawatzky, Phys. Rev. B **48**, 16929 (1993).

[5] L. A. Agapito, S. Curtarolo, and M. B. Nardelli, Reformulation of dft+u as a pseudohybrid hubbard density functional for accelerated materials discovery, Phys. Rev. X **5**, 011006 (2015).

[6] S. Coh and D. Vanderbilt, Python Tight Binding (PythTB) (2022).

[7] H. C. Kandpal, I. Opahle, Y.-Z. Zhang, H. O. Jeschke, and R. Valentí, Revision of Model Parameters for k-Type Charge Transfer Salts: An Ab Initio Study, Phys. Rev. Lett. **103**, 067004 (2009).

[8] J. Li, Z. Cao, J. Su, R. Wang, H. Li, Y. Nomura, X. Yang, and H.-K. Tang, *Ab initio* studies on interactions in k3c60 under high pressure, Physical Review B **109**, 134513 (2024).

[9] H. Shinaoka, T. Misawa, K. Nakamura, and M. Imada, Mott Transition and Phase Diagram of κ-(BEDT-TTF)$_2$ Cu(NCS)$_2$ Studied by Two-Dimensional Model Derived from *Ab initio* Method, Journal of the Physical Society of Japan **81**, 034701 (2012).

[10] Y. Kurosaki, Y. Shimizu, K. Miyagawa, K. Kanoda, and G. Saito, Mott Transition from a Spin Liquid to a Fermi Liquid in the Spin-Frustrated Organic Conductor kappa-$(ET)_2Cu_2(CN)_3$, Phys. Rev. Lett. **95**, 177001 (2005).
